# The prevalence of exotoxins, adhesion, and biofilm-related genes in *Staphylococcus aureus* isolates from the main burn center of Tehran, Iran 

**DOI:** 10.22038/ijbms.2019.34908.8291

**Published:** 2019-11

**Authors:** Zahra Mir, Narges Nodeh Farahani, Sara Abbasian, Faranak Alinejad, Mahboubeh Sattarzadeh, Ramin Pouriran, Mostafa Dahmardehei, Mehdi Mirzaii, Seyed Sajjad Khoramrooz, Davood Darban-Sarokhalil

**Affiliations:** 1 Microbial Biotechnology Research Center, Department of Microbiology, School of Medicine, Iran University of Medical Sciences, Tehran, Iran; 2 Burn Research Center, Shahid Motahari Hospital, Iran University of Medical Sciences, Tehran, Iran; 3 School of Medicine, Shahid Beheshti University of Medical Sciences, Tehran, Iran; 4 School of Medicine, Shahroud University of Medical Sciences, Shahroud, Iran; 5 Cellular and Molecular Research Center, Yasuj University of Medical Sciences, Yasuj, Iran

**Keywords:** Adhesin and biofilm genes, Burn, Iran, MRSA, Virulence factors

## Abstract

**Objective(s):**

The present study investigated the prevalence of genes encoding for exotoxins, adhesion and biofilm factors in *Staphylococcus aureus *isolates obtained from samples in a referral burn hospital in Tehran, Iran.

**Materials and Methods:**

*S. aureus* isolates obtained from patients, personnel and surfaces in the wards of a burn hospital were identified and confirmed by biochemical and molecular tests, respectively. The susceptibility of isolates was determined using the disk diffusion method. Virulence factors were detected by multiplex PCR.

**Results:**

The frequency of *hla*, *hlb*, *hld*, *hlg*, *tst* and *pvl* genes was 92.8%, 34.7%, 89.8%, 11.9%, 10.7%, and 0.5% respectively. The results revealed that the *hla* gene had the highest frequency among isolates (94.4% for methicillin-resistant *S. aureus *(MRSA) and 89.8% for methicillin-susceptible *S. aureus* (MSSA)). The most prevalent adhesion and biofilm-related gene was *eno* (85.6%). The prevalence of the remaining genes was as follows: *fib* (71.8%), *clfB* (70%), *cna* (59.2 %), *fnbB* (17.9%), *icaA* (72.4%), and *icaD* (85.6%). The incidence of *fib*, *hlb*, *hlg*, and *tst* genes was significantly higher in MRSA isolates compare to the MSSA isolates. Moreover, the resistance rates for all antibiotics were higher is MRSA isolates except for nitrofurantoin and chloramphenicol antibiotics.

**Conclusion:**

Data indicate the high prevalence rates of virulence factors among *S. aureus* isolates, especially MRSA strains in the burn hospital. This should to be taken into account in the development of an effective infection control policy and continuous monitoring of drug resistance in hospitals.

## Introduction

Damage to the skin barrier of patients with burn injuries increases the risk of microbial colonization, growth, and infection (1. Burn wound infections are a common dilemma in burn centers and are considered as a significant cause of mortality in burn patients. *Staphylococcus aureus* has been identified as a major etiological agent of infection in hospitalized burn patients (2). The following virulence factors have been identified for *S. aureus*: leukocidin (Panton-Valentine leukocidin; PVL), hemolysins (α, β, γ, δ), toxic shock syndrome toxin-1 (TSST-1), exfoliative toxins (ETs), and staphylococcal enterotoxin (SE) (3). 

The virulence factors of *S. aureus* have various effects on human health. Leukotoxins and hemolysins can affect biological membrane leading to cell death (4). PVL can lead to skin and soft tissue infections, necrotizing pneumonia, and necrotizing fasciitis (5). Bacterial attachment to host tissues is the primary stage of infection. At this stage, adherence of *S. aureus* is mediated by microbial surface component-recognizing adhesive matrix molecules (MSCRAMMs) (6) including fibronectin–binding proteins A and B (FnbA and FnbB), fibrinogen-binding proteins (Fib), collagen binding protein (cna), clumping factors A and B (clfA and clfB), and laminin binding protein (eno) (7). A clear *S. aureus* biofilm can be formed on damaged skin, mucosa, and artificial surfaces (8). Furthermore, products of the *ica *locus and polysaccharide intercellular adhesin (PIA) are critical for intercellular bacterial adherence and biofilm formation (9).

Studies show that the epidemiology and virulence factors of *S. aureus* strains in hospitals, particularly in burn centers, are a challenge for infection control programs (10). Environmental surfaces and healthcare personnel are the leading sources of the spread of pathogens causing nosocomial infections. Early identification of *S. aureus* isolates obtained from patients, personnel and surfaces in hospitals can help us determine important virulence factors of the isolates for a more efficient infection control. The aim of this study was to investigate the prevalence of genes encoding for exotoxins, adhesion, and biofilm factors in *S. aureus *isolates in a burn hospital in Tehran, Iran. 

## Materials and Methods

***Sample collection and identification of bacterial isolates***

This cross-sectional study was conducted on samples obtained from Shahid Motahari Hospital (the main specialized burn center in Tehran, Iran) from December 2015 to December 2016. Samples were obtained from hospital personnel (both nostrils) and surfaces (beds, Ambu bags, door knobs, medical trolleys, chairs, suction, etc.). Samples were collected from personnel three times using wet sterile swabs and from surfaces monthly during the study period. All samples were cultured on brain-heart infusion media. Burn wound swabs were also taken as part of the routine screening for MRSA during the study period. Biochemical tests (mannitol salt agar media, susceptibility to bacitracin, catalase, DNase and tube coagulase tests, mannitol fermentation) were performed for bacterial identification.

***Antimicrobial susceptibility tests***

Antibiotic susceptibility was determined using the standardized Kirby-Bauer disc diffusion method on Mueller-Hinton agar. The antimicrobial agents tested included nitrofurantoin (300 µg), gentamicin (10 μg), mupirocin (20 μg), rifampicin (5 μg), norfloxacin (10 μg), tigecycline (15 μg), trimethoprim-sulfamethoxazol (25 μg), cefoxitin (30 μg), chloramphenicol (30 μg), erythromycin (15 μg), clindamycin (2 μg), tetracycline (30 μg), penicillin (10 units), linezolid (30 μg), synercid (quinupristin/dalfopristin; 15 μg), and imipenem (10 μg). Erythromycin-induced clindamycin resistance was determined using the disk approximation test. The isolate with cefoxitin resistance was MRSA. *S. aureus *ATCC 25923 was used as the control for sensitivity testing.

***DNA extraction and molecular identification of MRSA isolates***

DNA of *S. aureus* was extracted using the boiling method as described previously (11). For confirmation of *S. aureus* identification and determination of methicillin resistance, all isolates were subjected to the *S. aureus-*specific nuclease (*nuc*A*) *and* mec*A-specific PCR (12, 13). 

***Detection of exotoxin- and biofilm-related genes***

Multiplex PCR was used for the detection of virulence factors- encoding genes including *pvl*, *tst* (toxic shock syndrome toxin-1- encoding gene), and *hla*, *hlb*, *hld* and *hlg* genes (hemolysin-encoding genes). The following MSCRAMMs were detected using specific primers: clumping factor B (*clfB*), fibronectin-binding protein (*fnbB*), collagen-binding protein (*cna*), lamina-binding protein (*eno)*, fibrinogen-binding protein (*fib*), and biofilm-encoding genes (*icaA* and *icaD*) (7,12-16). The PCR products (3 μl) were run on 1.5% agarose gel and stained with SYBR® Safe DNA stain. Electrophoresis of PCR products was carried out in 0.5×TBE buffer for 90 min at 110 mV. The standard PCR conditions and primers used for the multiplex PCR reactions in this study are listed in Table 1 and Table 2, respectively. The results of antibiotic susceptibility testing and the detection of virulence genes among *S. aureus* isolates were analyzed by Pearson Chi-Square and Fisher’s tests.

## Results

***Bacterial isolates***

In the present experimental study, from a total of 167 *S. aureus *isolates, 108 (65%) were identified as MRSA (79/123 isolates obtained from patients, 22/30 from surfaces, 7/14 from personnel), while 59 (35%) were identified as MSSA (44/123 from patients, 8/30 from surfaces and 7/14 from personnel).

***Antimicrobial susceptibility testing***

**Figure 1:**
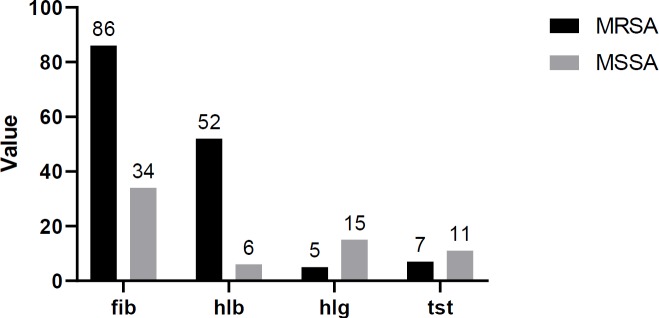
The results of statistical analysis on virulence genes among MRSA and MSSA isolates

MRSA: methicillin resistant *Staphylococcus aureus* ; MSSA: methicillin-susceptible *Staphylococcus aureus*

The antimicrobial resistance rate in *S. aureus* isolates to penicillin was 78%, imipenem 69%, cefoxitin 65%, norfloxacin 61%, erythromycin 59%, gentamicin 58%, tetracycline 57%, mupirocin 57%, clindamycin 54%, rifampicin 44%, trimethoprim-sulfamethoxazole 28%, tiecoplanin 9%, chloramphenicol 2% and nitrofurantoin 1%. The MRSA isolates revealed a significantly higher rate of antimicrobial resistance than the MSSA isolates (Table 3). The highest incidence of drug resistance in MRSA isolates was to penicillin (100%), imipenem (100%), cefoxitin (100%), norfloxacin (87%), and gentamicin (86%). All isolates were susceptible to quinupristin-dalfopristin, linezolid, and tigecycline (Table 3). Statistical analysis of antibiotic susceptibility patterns in MRSA and MSSA isolates are shown in Figure 1. Results show that resistance to all antibiotics (except for chloramphenicol and nitrofurantoin) was significantly higher in MRSA isolates compared to the MSSA isolates.

***Exotoxins and adhesin genes***

The frequency of *hla*, *hlb*,* hld*,* hlg*,* tst, *and *pvl* genes was 92.8%, 34.7%, 89.8%, 11.9%, 10.7%, and 0.5%, respectively. Results revealed that the *hla* gene was the most frequent gene among isolates (94.4% for MRSA and 89.8% for MSSA). The frequency of other toxin genes in the MRSA and MSSA isolates respectively was 91.6% and 86.4% for *hld,* 48.1 and 10.1% for *hlb*, 6.4% and 18.6% for *tst *and 4.6% and 25.4% for *hlg*. The *pvl* gene was detected in 1.6% of MSSA isolates, but was not detected in MRSA. 

Among the adhesion genes, the most prevalent was *eno *(85.6%). The incidence of other genes were as follows: *fib* (71.8%)*, clfB *(70%),* cna *(59.2%) and *fnbB* (17.9%). The frequency of these genes in MRSA isolates was 87% for *eno*, 79.6% for* fib*, 67.5% for* clfB*, 61.1% for *can, *and 18.5% for *fnbB.* The *clfB* gene was detected in MSSA isolates at a significantly higher rate (7%) compared to the MRSA isolates; *icaA,* and *icaD* were positive in 72.4% and 85.6% of isolates, respectively. The *icaD *gene was found at analgesic rates in MRSA (85.1%) and MSSA (86.4%) isolates. The frequency of the *icaA* gene was slightly higher in MRSA (76.8%) than in MSSA (64.4%) isolates. 

**Figure 2:**
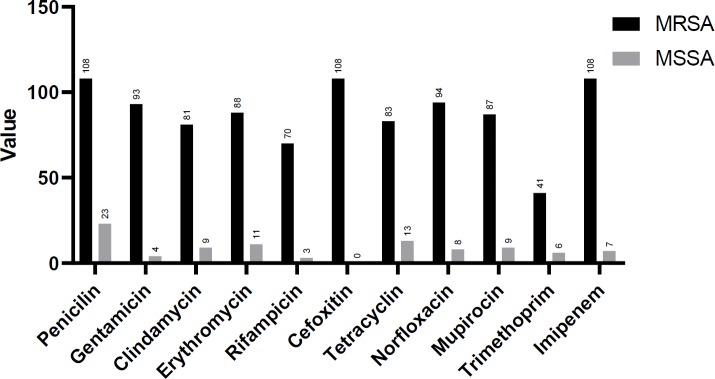
The results of statistical analysis on antibiotic susceptibility tests among MRSA and MSSA isolates

Overall, no significant difference in terms of virulence genes was found between the MRSA and MSSA isolates. The rates of genes detected from patients, surfaces and personnel are shown in Table 4. The coexistence of adhesion factors-related genes was detected in 8.9% of patient and 10% of surfaces isolates. Both the *icaA* and *icaD* genes were detected in 83.3%, 57.7%, and 57.1% of isolates from surfaces, patients and personnel, respectively. The antibiotic resistance profile and gene combination patterns in the MRSA isolates are shown in Table 5. None of the isolates showed the coexistence of toxin genes. Statistical analysis of the distribution of virulence genes among MRSA and MSSA isolates is shown in Figure 2. The results show that the incidence of *fib, hlb, hlg,* and *tst* genes was significantly higher in MRSA isolates compared to the MSSA isolates.

## Discussion

In the present study, a high prevalence of MRSA (65%) was found in samples obtained from a burn hospital in Tehran, Iran (from patients, healthcare personnel and surfaces). These results are in accordance with the results of other studies from Iran and Bangladesh that reported a high frequency of MRSA in burn patients (17-24). In contrast, Darban-Sarokhalil *et al.* reported a lower frequency of MRSA in two Iranian hospitals (11). The results of the present study indicated a lower prevalence of MRSA compared to another study in Uganda in which 100% of the isolates obtained from burn units were found to be MRSA (21). These discrepancies could be attributed to different infection control criteria, antibiotic administration, study design and laboratory testing for determination of methicillin resistance. 

In the present study, there was a significant increase in the rate of resistance to antibiotics such as penicillin, tetracycline, erythromycin, gentamycin, clindamycin, mupirocin, and rifampicin in MRSA isolates. Data suggest the possibility of multiple antimicrobial resistance in hospital strains. This could be due to the continuous and empirical usage of broad-spectrum antibiotics and the absence of a suitable antibiotic treatment policy (23, 25). Despite the use of vancomycin and linezolid for the treatment of life-threatening infections caused by resistant *S. aureus* strains, all isolates were susceptible to new drugs (quinupristin-dalfopristin, linezolid, and tigecycline). These results are in accordance with those of Bayat *et al* (26). In the current study, the overall rate of resistance to mupirocin in MRSA isolates was 81%. Mupirocin resistance rate in MRSA isolates obtained from personnel, patients, and surfaces was 100%, 78%, and 82%, respectively. Chen *et al.* (2) reported high incidence of mupirocin resistance in most MRSA isolates in burn centers. The widespread use of mupirocin for prolonged periods, particularly for decolonization of healthcare personnel, bedsores and other skin lesions could be associated with the development of mupirocin resistance (27, 28).

In addition to antibiotic resistance, another factor that prevents effective treatment of staphylococcal infections in burn patients is biofilm formation (18). The importance of biofilm formation is unique in the medical world. Notably, bacterial species present in biofilms display more resistance to antibiotics and disinfectants (29). In burn wounds, molecules such as collagen, ﬁbronectin, ﬁbrinogen and other factors are present at the wound surface. *S. aureus* encodes many proteins that specifically interact with human cellular matrix components enabling the microorganism to colonize burn wounds (19). In our study, the frequency of *eno, clfB,* and *cna* genes was significantly higher than another study by Motallebi *et al* (30). 

Another virulent factor that contributes to biofilm formation is PIA which can be encoded by the *ica* ADBC operon. Of the *ica* genes, *icaA* and *icaD* play an eminent role in biofilm production by *S. aureus* (28). Results show that* icaA* and *icaD* genes were present in 76.8% and 85.1% of isolates, respectively. Table 5 shows that the most indispensable genes detected in the MRSA isolates were identified as *icaA+icaD* followed by *icaA*+*icaD*+*hla*+*hld*. The frequencies obtained in the current study were significantly higher than those obtained in other studies performed in Iran (28). Satorres *et al.* (31) reported the frequencies of *icaA* and *icaD* genes in *S. aureus* isolates obtained from the hospital staff that were lower than those reported in the current study. The diversity of the prevalence of biofilm-encoded genes could be related to the variety of bacterial strains at different geographical regions.

Hemolysins (alpha, beta, delta and gamma) and PVL are able to damage host cells by their cytolytic effects. TSST-1 has been associated with several acute or chronic human diseases, including TSST (32). In the present study, the frequency of the *hla* and *hld* genes were 92.8% and 89.8%, respectively. This is in accordance with the results of Kateete *et al.* (21) in Uganda who reported a frequency of 100% for these genes. It was revealed that the frequency of the coexistence of *hla*+*hld* genes in *S. aureus* isolates obtained from patients, surfaces and personnel was 84.5%, 90% and 92.8%, respectively. High rates (93.6% and 88.6%) were recorded for patient-derived MRSA isolates harboring *hla* and* hld *genes*, *respectively. A similar rate for *hla* and *hld* were discovered in burn patients by Rodrigues *et al* (10). While *hla* and *hld *genes were found in all surface-derived MSSA isolates by Gharsa *et al.* (33), in the current work, these genes were detected in 87.5% and 75% of surface-derived MSSA isolates, respectively.

In the current study, the* hlb* and *hlg* genes were detected in 48.1% and 4.6% of the isolates, respectively. The rate of *hlb *(43%) in patient-derived MRSA isolates was similar to the study conducted by Karmakar *et al.* (34); however, this rate was lower than that found by Liu *et al* (35). The frequency of *hlb* gene in MRSA isolate obtained from personnel and surfaces (57.1% and 63.3%, respectively) was higher than those isolates obtained from patients. The *hlg* gene was detected in 6.3% of MRSA isolates. Diversity in the prevalence of hemolysin-, adhesion- and biofilm- encoding genes can be associated with the diversity of bacterial strains in different geographical areas. 

A key virulence factor in *S. aureus* infections, especially in skin and soft-tissue infections is the PVL. This toxin has been recognized as a virulence factor associated with tissue necrosis (36). Data regarding the danger of infections caused by PVL-producing MRSA strains have raised public health concerns (5). In the current study, no *pvl* positive MRSA isolate was detected. This could be attributed to the fact that *pvl* is more related to community acquired MRSA strains. These findings were similar to the findings of Mkrtchyan *et al* (37). In contrast, a study from Brazil found that 14.6% of MRSA isolates had the *pvl *gene (10) . A study conducted in England reported that 2% of clinical *S. aureus* isolates (MRSA and MSSA) harbored the *pvl* gene (5). In the current study, only one patient-derived MSSA isolate (2.2%) was positive for *pvl gene*. Murray *et al*. (38) reported that *pvl* was detected in one MRSA isolate obtained from burn patients.

The frequency of the *tst* gene reported in Germany (39), Iran(40) and Korea(41) was 14%, 26.41% and 72.2%, respectively. In the current study, the frequency of the *tst* gene was 10.7%. Kateete *et al.* (21) studied patients, healthcare workers and surfaces in the burn units in a hospital. The rate of *hla*, *hld,* and *tst* in their study were analogous with those from the present study, but *hlg* and *pvl* genes were detected at higher frequencies than in the current study. Gharsa *et al.* (33) detected MSSA isolates on hospital surfaces and found *tst* in 60% of isolates; however, *tst* was not detected in MSSA isolates from the surfaces in the present study. The rate of *tst* in patient-derived MSSA isolates (25%) was higher than in patient-derived MRSA isolates (6.3%), which is similar to the results of a study by Liu *et al* (35). De Boeck *et al.* (42) found the prevalence of *tst* and *pvl* genes in the isolates obtained from healthcare workers to be 17.5% and 28.5%, respectively. In the present study, these genes were not detected in personnel. In fact, the difference in incidence could be related to the variation in the geographical area and the origin of the strains. 

**Table 1:** Primers and product size of PCR for detection of the exotoxins and biofilm genes

Genes	Sequence (5-3)	Product size (bp)	Reference
*cna* *eno* *fnbB* *fib* *clfB* *icaA* *icaD* *hla* *hlb* *hld* *hlg* *pvl* *tst* *nuc* *mecA *	F-GTCAAGCAGTTATTAACACCAGAC R-AATCAGTAATTGCACTTTGTCCACTG F-ACGTGCAGCAGCTGACT R-CAACAGCATYCTTCAGTACCTTC F-GTAACAGCTAATGGTCGAATTGATACT R-CAAGTTCGATAGGAGTACTATGTTC F-CTACAACTACAATTGCCGTCAACAG R-GCTCTTGTAAGACCATTTTCTTCAC F-ACATCAGTAATAGTAGGGGGCAAC R-TTCGCACTGTTTGTGTTTGCAC F-CCTAACTAACGAAAGGTAG R-AAGATATAGCGATAAGTGC F_AAACGTAAGAGAGGTGG R-GGCAATATGATCAAGATAC F- CTG ATT ACT ATC CAA GAA ATT CGA TTG R- CTT TCC AGC CTA CTT TTT TAT CAG T F- GTG CAC TTA CTG ACA ATA GTG C R- GTT GAT GAG TAG CTA CCT TCA GT F-AAG AAT TTT ATC TTA ATT AAG GAA GGA GTG R- TTA GTG AAT TTG TTC ACT GTG TCG A F- GTC AYA GAG TCC ATA ATG CAT TTA A R- CAC CAA ATG TAT AGC CTA AAG TG F-ATCATTAGGTAAAATGTCTGGACATGATCCA R-GCATCAASTGTATTGGATAGCAAAAGC F- ACCCCTGTTCCCTTATCATC R- TTTTCAGTATTTGTAACGCC F-CTGGCATATGTATGGCAATTGTT R-TATTGACCTGAATCAGCGTTGTCT F-GTGAAGATATACCAAGTGATT R-ATGCGCTATAGATTGAAAGGAT	423 302 524 404 205 1315 381 209 309 111 535 433 326 664 147	15

**Table 2:** Cycles and condition of multiplex PCRs in this study

Genes	Cycles of amplification	Initial denaturation	Denaturation	Annealing	Extension	Final extension	Reference
*nucA, mecA*	30	5min at 94	45s at 94	45s at 57	1min at 72	5min at 72	12, 13
*cna, eno, fib, fnbB, clfB*	25	5min at 94	1 min at 94	1min at 55	1min at 72	10 min at 72	
*icaA, icaD *	30	5min at 94	45s at 92	45s at 49	1min at 72	7min at 72	
*hla, hld, hld, hlg*	30	5min at 94	45s at 94	45s at 57	1min at 72	5min at 72	
*tst*	35	5min at 94	2min at 94	2min at 57	1min at 72	7min at 72	
*pvl*	30	5min at 95	40s at 95	40s at 54	45s at 72	5min at 72	

MRSA: methicillin resistant *Staphylococcus aureus*; MSSA: methicillin-susceptible *Staphylococcus aureus*

**Table 3:** Antibiotics resistance in MRSA and MSSA strains in patients, surfaces and personnel in burn Shahid Motahari Hospital, Tehran, Iran

Antibiotics	Patients N (%)		Surfaces N (%)		Personnel N (%)	
	MRSA(79)	MSSA(44)	MRSA(22)	MRSA(7)	MSSA(8)	MSSA(7)
Penicillin	79 (100)	16 (36)	22 (100)	4 (50)	7 (100)	3(43)
Gentamicin	67 (85)	2 (5)	21 (95)	2 (25)	5 (71)	0
Clindamycin	62 (78)	7 (16)	18 (82)	2 (25)	1 (14)	0
Erythromycin	68 (86)	9 (20)	18 (82)	2 (25)	2 (29)	0
Nitrofurantoin	1 (1)	0	0	1(13)	0	0
Rifampicin	48 (61)	3 (7)	17 (77)	0	5 (71)	0
Quinupristin- Dalfopristin	0	0	0	0	0	0
Linezolid	0	0	0	0	0	0
Tigecycline	0	0	0	0	0	0
Cefoxitin	79 (100)	0	22 (100)	0	7 (100)	0
Tetracycline	61 (77)	11(25)	18 (82)	2 (25)	4 (57)	0
Norfloxacin	68 (86)	6 (14)	20 ( 91)	2 (25)	6 (86)	0
Mupirocin	62 (78)	6 (14)	18 (82)	2 (25)	7 (100)	1(14)
Trimethoprim- Sulfamethoxazole	32 (41)	6 (14)	8 (36)	0	1 (14)	0
Imipenem	79 (100)	4 (9)	22 (100)	3 (38)	7 (100)	0
Chloramphenicol	3 (4)	1 (2)	0	0	0	0

MRSA: methicillin resistant *Staphylococcus aureus*; MSSA: methicillin-susceptible *Staphylococcus aureus*

**Table 4:** Distribution of virulence genes in *Staphylococcus aureus*, MRSA and MSSA isolates of patients, surfaces and personnel in burn Shahid Motahari Hospital, Tehran, Iran

Virulence genes	Patients (123)		Surfaces (30)		Personnel(14)	
	MRSA (79) N (%)	MSSA (7) (79) N (%)	MSSA (44) (79) N (%)	MRSA (22) (79) N (%)	MSSA (8) (79) N (%)	MRSA (7) (79) N (%)
**Adhesion**						
*eno*	65 (82.2)	35 (79.5)	22 (100)	7 (87.5)	7 (100)	7 (100)
*cna*	45 (56.9)	26 (59)	18 (81.8)	4 (50)	3 (42.8)	3 (42.8)
*clfB*	49 (62)	34 (77.2)	19 (86.3)	7 (87.5)	5 (71.4)	3 (42.8)
*fib*	59 (74.6)	25 (56.8)	20 (90.9)	5 (62.5)	7 (100)	4 (57.1)
**Toxin **						
*hla*	17 (21.5)	7 (15.9)	2 (9)	3 (37.5)	1 (14.2)	0
*hlb*	74 (93.6)	40 (90.9)	21 (95.4)	7 (87.5)	7 (100)	6 (85.7)
*hld*	34 (43)	5 (11.3)	14 (63.6)	1 (12.5)	4 (57.1)	0
*hlg*	70 (88.6)	38 (86.3)	22 (100)	6 (75)	7 (100)	7 (100)
	5 (6.3)	13 (29.5)	0	1 (12.5)	0	1 (14.2)
*pvl*	5 (6.3)	11 (25)	2 (9)	0	0	0
**Biofilm**						
*tst*	0	1 (2.2)	0	0	0	0
*icaA*	56 (70.8)	28 (63.6)	21 (95.4)	6 (75)	6(85.7)	4 (57.1)
*icaD*	64 (81)	37 (84)	21 (95.4)	8 (100)	7(100)	6 (85.7)

MRSA: methicillin resistant *Staphylococcus aureus*; MSSA: methicillin-susceptible *Staphylococcus aureus*

**Table 5:** Antibiotic resistance profile and gene combination patterns in MRSA isolates of burn hospital

Resistance profile	Gene combination		
	*icaA+icaD*	*icaA+icaD+hla+hld*	*icaA+icaD+hla+hld+eno+fib*
GM, MUP, RP, NOR, E, CD	36 (33%)	33 (30.5%)	25 (23.1%)
PG, IMI, T, E, CD	46 (42.5%)	42 (38.5%)	40 (37%)
GM, MUP, NOR, RP	44 (40.7%)	43 (39.8%)	35 (32.4%)
MG, MUP, RP, NOR, E, CD, T, PG, IMI	33 (30.5%)	31 (28.5%)	25 (23.5%)

PG, Penicillin; MG, Gentamicin; CD, Clindamycin; E, Erythromycin; RP, Rifampicin; T, Tetracycline; NOR, Norfloxacin; MUP, Mupirocin; IMI, Imipenem;

Hospital environments play an important role in the transmission of MRSA and the development of infection in patients (10). In the current study, the results of antibiotic susceptibility patterns and virulence factors, especially *pvl*, indicate that a potential outbreak in hospitals could be associated with the personnel or the surfaces. Colonized healthcare personnel and environmental sources could serve as a reservoir and disseminator of MRSA in hospitals. Therefore, using proper disinfectant and regular screening for MRSA among healthcare workers and patients, in addition to improved precautions for personnel are essential for infection control (17, 43). Moreover, methods in molecular epidemiology are compulsory for the continuous surveillance and rapid identification of prevalent strains of *S. aureus* and MRSA clones. These methods have been shown to contribute to the control of the spread of bacterial infections in healthcare settings (11, 20, 44). 

## Conclusion

It was determined that the high prevalence of virulence factors and the elevated rate of antibiotic resistance among isolates obtained from patients, personnel and surfaces of burn hospital necessitate proper implementation of an effective infection control policy and continuous monitoring for drug resistance.

## Conflicts of Interest

All contributing authors declare no conflicts of interest.
